# FEM-Based Study of Precision Hard Turning of Stainless Steel 316L

**DOI:** 10.3390/ma12162522

**Published:** 2019-08-08

**Authors:** Ahmed Elkaseer, Ali Abdelaziz, Mohammed Saber, Ahmed Nassef

**Affiliations:** 1 Department of Production Engineering and Mechanical Design, Faculty of Engineering, Port Said University, Port Fuad 42526, Egypt; 2 Institute for Automation and Applied Informatics, Karlsruhe Institute of Technology, 76344 Eggenstein-Leopoldshafen, Germany; 3 Northern Workshop, Port Said Shipyard, Suez Canal Authority, Port Fuad 42526, Egypt; 4 Department of Mechanical Engineering, College of Engineering, King Faisal University, Al-Ahsa 31982, Saudi Arabia; 5 High Institute of Engineering & Technology, North Sinai, EL-Arish 45511, Egypt

**Keywords:** precision hard turning, chip formation, surface quality, minimum chip thickness, cutting edge radius, FEM

## Abstract

This study aims to investigate chip formation and surface generation during the precision turning of stainless steel 316L samples. A Finite Element Method (FEM) was used to simulate the chipping process of the stainless steel but with only a restricted number of process parameters. A set of turning tests was carried out using tungsten carbide tools under similar cutting conditions to validate the results obtained from the FEM for the chipping process and at the same time to experimentally examine the generated surface roughness. These results helped in the analysis and understanding the chip formation process and the surface generation phenomena during the cutting process, especially on micro scale. Good agreement between experiments and FEM results was found, which confirmed that the cutting process was accurately simulated by the FEM and allowed the identification of the optimum process parameters to ensure high performance. Results obtained from the simulation revealed that, an applied feed equals to 0.75 of edge radius of new cutting tool is the optimal cutting conditions for stainless steel 316L. Moreover, the experimental results demonstrated that in contrast to conventional turning processes, a nonlinear relationship was found between the feed rate and obtainable surface roughness, with a minimum surface roughness obtained when the feed rate laid between 0.75 and 1.25 times the original cutting edge radius, for new and worn tools, respectively.

## 1. Introduction

One of the important recent milestones in the machining field has been the development of advanced precision machining processes. Chief among them, precision turning is an efficient process for machining precise cylindrical components with tight tolerances and high quality features, which has increased its deployment for industrial applications [1]. Precision hard turning refers to the use of a single point tool to machine materials that have high strength, corrosive resistance, toughness, ductility, and wear resistance [2]. These are referred to as ‘hard-to-cut’ materials, e.g., nickel superalloys, titanium alloys, and stainless steel [3,4]. Precision hard turning is considered a profitable and dependable alternative to grinding, with a reduction in machining time as high as 60% [5]. It is worth emphasizing that appropriate cutting conditions have to apply to enable precision hard turning to deliver very fine machined surfaces [6,7,8].

Precision hard turning is used in numerous applications, including bearings, dies, gears, and molds because it improves the quality of the product while simultaneously reducing lead times and manufacturing costs [9]. These benefits are especially noticeable with, for example ceramics, polycrystalline cubic boron nitride (PcBN) and tungsten carbide tools which are commercially available as super-hard tool materials. Different approaches, entailing experimental [1,3,4,5,6,7,9,10,11,12,13], analytical [14,15], and FEM [16,17,18,19,20,21,22,23,24,25,26,27,28,29], have been utilized to examine the performance of a wide range of machining operations in terms of surface quality, generated cutting force and tool wear. Nevertheless, the potential of the precision hard turning process has not been fully realized as of yet. This is caused because of the constraints of size-scale of the process. As in precision hard turning, the cutting edge radius of the tool and the feed rate are comparable in scale. Therefore, there can be substantial differences between the physical principles that govern the underlying phenomenon of this technique at the macro scale. Resulting in undesirable changes to the chip formation and surface generation processes [10,11]. In particular, in contrast to conventional turning where the obtainable surface roughness decreases proportionally with the reduction of undeformed chip thickness (applied feed), in precision hard turning the undeformed chip thickness can be smaller than the cutting edge radius of the tool [12,16]. When the undeformed chip thickness is less than the minimum chip thickness, a certain thickness of the material to be removed under which no cutting mechanism occurs, ploughing is the governing mechanism. This is associated with generation of rough surface. In addition, the ploughed material ahead of the tool cutting edge exerts high stresses on the cutting tool which accelerates tool wear and results in even higher surface roughness [12,17].

A number of researchers have performed FEM simulation of machining processes validated by experimental tests, with the aim of determining the optimum parameters for a given process [18].

Shao et al., [19] investigated tool wear and cutting temperature for the turning of Ti-6Al-4V alloy with a Tungsten carbide–cobalt cutting tool. Combining FEM with thermo-dynamics, a comparison was undertaken between experimental results and simulation predictions for depth of tool wear with cutting temperature. The accuracy of the FEM was confirmed by good match of practical results and FEM model simulations, but only on condition that the parameters input for cutting tool and workpiece were correctly chosen.

Akbar et al., [20] examined the performance of the machining process in terms of the heat generated and its division between the chip and the cutting tool. The investigation was carried out by FEM modelling and, then, was validated by experiments with infrared techniques used to measure the cutting temperature. It was reported that the heat generated could significantly alter the contact area between tool and chip, and therefore affect the ability of FEM model to simulate the process. Accurate measurement of the heat division between chip and tool was necessary for accurate FEM modelling of the cutting operation.

Zhou et al., [21] used a polycrystalline diamond (PCD) tool to carry out 2-D orthogonal cutting of SiCp/Al composites. The study investigated von-Mises equivalent stresses and the cutting force under different cutting conditions both experimentally and with a FEM model. Simulation and experimental results agreed that the machining of SiCp/Al composites needs to be at as high cutting speed as possible. However, they found that the inverse applied to cutting depth. They also found that the removal of SiCp particles was dependent on the relative position of cutting tool and particle.

Li, [22] used FEM simulations to simulate the progression of tool wear in turning operation under conditions where cutting process variables were difficult to obtain experimentally.

Yang et al., [23] used FEM to investigate the mechanical behavior of hydrogenated-6Al-4V alloy at high strain rates and elevated temperatures using a split Hopkinson pressure bar. The cutting process was modelled numerically, and the results obtained showed that an increase in hydrogen content significantly affected the temperature and cutting forces. Further investigation by simulation showed that it was better to machine titanium hydride at high cutting speeds.

John et al., [24] investigated surface roughness of AISI 1020 steel when cut at different speeds with different feed rates and depths of cut using HSS tools and CNMA diamond inserts. This study used both FEM simulation and experimental tests. The differences between the experimental results and simulation results ranged between 3.74% and 22.8% for the HSS tool and between 1.15% and 11.8% for the CNMA insert. The best surface quality for the AISI 1020 steel using the CNMA insert tool was obtained at: cutting speed of 5.45 mm/sec, depth of cut of 0.50 mm and feed rate of 0.05 mm/rev. However, the quality of the surface obtained using diamond insert was better than that obtained using the HSS tool.

Haddag et al., [25] reported a two-step modelling strategy to investigate cutting tool heat transfer. The initial step was to simulate chip formation and estimate the cutting forces via a 3-D thermo-mechanical FEM model. The final step was to simulate the thermal response of the cutting tool under thermal load using a 3-D FEM thermal model. This thermal load was estimated using quantities gained during the initial step; including sliding velocity, and contact area and pressure. Comparison between model and experimental results were in good agreement.

Ali et al., [26] compared the results obtained from an FEM model with experimental results when studying the machining of Ti-6Al-4V. However, the simulation and experimental results showed a significant difference.

Bushlya et al., [27] developed an FEM model to simulate the creation of machined subsurface layer when turning using a tool with a nose-radius and compared the simulation results with experimental measurements. The simulation predicted multiple deformations of the workpiece material would take place in the region around the tip of any tool with a nose radius. Numerous distortions of the machined surface were observed when machining at low feed rates using tools with a sizeable nose radius. These results suggested that the formation of the machined surface was, in significant part, due to severe wear of the cutting tool which was the result of work-hardening of the surface material.

Aurich et al., [28] developed an FEM model to analyze the accuracy of turning operations using dry machining and compared the simulated results with experiments. It was shown that the cutting operation had a substantial effect on the heat generated which then affected the cutting tool, tool holder and workpiece, and thus could detract from the accuracy of the process. It was claimed that least depth of cut, maximum cutting speed, and feed rate, gave maximum accuracy of machining. These authors found that the temperatures of both tool holder and tool must be considered to ensure maximum accuracy of machining.

Maruda et al., [30] utilized FEM to study chip formation and tool wear of sintered carbide P25 tool when turning AISI 1045 steel under different cooling methods, i.e., dry machining, MQCL method and MQCL + EP/AW, under a range of cutting speeds. The results revealed that MQCL method with phosphate ester-based additive reducing the friction coefficient between the chips and rake face of the tool which and chip thickening coefficient which eases the chip removal away from the cutting zone.

From the literature it is clear that there has been a noticeable number of studies of precision hard turning. Nevertheless, there is still a need for a deeper understanding of the science behind the underpinning chip formation and surface generation of the precision hard turning process at micro scale. This is because the large body of the reported work has, generally, examined the process responses at conventional ranges of the process parameters (viz. when the values of the applied feed rate are well above the value of the cutting edge radius of the tool at which the underlying mechanism is mainly pure cutting) [1,4,7,31,32,33,34]. However, at micro-scale machining, to achieve a proper chipping mechanism and high-quality machined surface and minimum cutting forces, extremely restrictive cutting conditions, especially the applied feed, have to be identified and rigorously applied. Accordingly, the current research focusses on filling the gaps in the current scientific understanding of precision hard turning to examine the influence of the restricted cutting conditions (particularly applied feed rate at the vicinity of the edge radius of the cutting tool, due to their significant influence on the process performance). However, powerful engineering tools such as FEM are needed to better understand the process in its details. In this regard, the aim for this manuscript is to carry out a FEM-based study to examine the influence of a limited range process conditions (under which the cutting mechanism changes dramatically) on the chip formation and surface generation process when machining stainless steel 316L workpieces in a precision hard turning operation. The results of the FEM will be used to identify the optimum cutting parameters that enable proper chipping mechanism, higher quality of the machined surface and increased productivity of the process.

Following to this introduction, the remainder of this paper is organized as follows. Firstly, the FEM simulation procedure is described. Then, the experimental set-up is detailed, including a description of workpiece material, machine setup, applied cutting conditions and instrumentation. After that, the paper discusses the FEM obtained results, the agreement between these results and experiments. Next, the effect of the applied feed rate on surface quality is analyzed. Finally, the paper summarizes and draws relevant conclusions based on the results and discussion.

## 2. Modelling and Simulation

For the simulation processes, a commercial FEM package, Abaqus/explicit (version 6.14, Dassault Systèmes Simulia Corp., Providence, RI, USA), was used. A proposed analysis program was developed to simulate the chip formation process of a precision turning operation of stainless steel 316L. The results obtained from the FEM simulations were then compared to those obtained from the experimental work.

### 2.1. Material Models for the Cutting Process

The Johnson–Cook plasticity model is suitable for predicting deformation at high-strain rates and is particularly suitable for use with metals. It is commonly used to describe flow stress in metals as the product of temperature effects, strain and strain rate effects as given in Equation (1).

In this research, the chip formation in precision turning process of stainless steel 316L is studied where the applied depth of cut is 10 µm and the maximum feed rate is 70 µm. With this small feed rates and depth of cut, the effect of temperature on chip formation can be suppressed. Therefore, the value of temperature-dependent term Johnson–Cook equation was taken as unity which simplifies the calculations and accelerates the simulation.

(1) $$\sigma =\left(A+B{\left(\varepsilon_{pl}\right)}^{n}\right)\left[1+\mathrm{CLn}\left(\frac{{\dot{\varepsilon }}_{pl}}{{\dot{\varepsilon }}_{ref}}\right)\right]\left[1-{\left(\frac{T-T_{tr}}{T_{melt}-T_{tr}}\right)}^{m}\right]$$

where σ is the flow stress (MPa), *ε_pl_* is the equivalent plastic strain at which σ is calculated, ${\dot{\varepsilon }}_{pl}$ is the plastic strain rate (s^−1^), ${\dot{\varepsilon }}_{ref}$ is the reference plastic strain rate, which is generally normalized to a strain rate of 1 per second. *T* is the current analysis temperature (°C), *T_tr_* is the transient temperature (°C) which is defined as the temperature at, or below which, there is no temperature dependence on the expression of the flow stress. *T_melt_* (°C) is the melting temperature of the material.

A, B, C, n, and m are material constants of the workpiece and cutting tool that can be determined experimentally at or below the transition temperature. The values of these constants for the workpiece material (stainless steel 316L) are listed in Table 1.

Because the material is removed with chip formation, we use the Johnson–Cook damage model. This model assumes the plastic strain at the inception of damage, $\varepsilon_{D}^{pl}$, can be written as:

(2) $$\varepsilon_{D}^{pl}=\left[d_{1}+d_{2}\exp\left(d_{3}\frac{p}{q}\right)\right]\left[1+d_{4}Ln\left(\frac{{\dot{\varepsilon }}_{pl}}{{\dot{\varepsilon }}_{ref}}\right)\right]\left[1-d_{5}{\left(\frac{T-T_{tr}}{T_{melt}-T_{tr}}\right)}^{m}\right]$$

where $\varepsilon_{D}^{pl},$ is the equivalent plastic strain at the onset of damage, d_1_–d_5_ are failure parameters, p is the mean (or hydrostatic) stress, q is the von Mises stress, ${\dot{\varepsilon }}_{ref}$ is the reference strain rate, ${\dot{\varepsilon }}_{pl}$ is plastic strain rate, *T* is the current analysis temperature, *T_tr_* is the transient temperature, *T_melt_* is the melt temperature and m is a material constant.

In this study the effect of temperature on the machining process was suppressed (by setting d_5_ = 0.0 and m = 0) for two reasons, first due to the lack of material properties at different temperatures for both the workpiece and the cutting tool, and to simplify the calculations. The values of the parameters of Johnson–Cook damage model for stainless steel 316L are given in Table 1.

### 2.2. Materials: Mechanical and Physical Properties

Table 2 and Table 3 give the mechanical and physical properties of the stainless steel 316L. It needs to be noted that the accuracy of the obtained results depends on the accuracy of this data.

### 2.3. FEM Mesh

**Figure 1:**
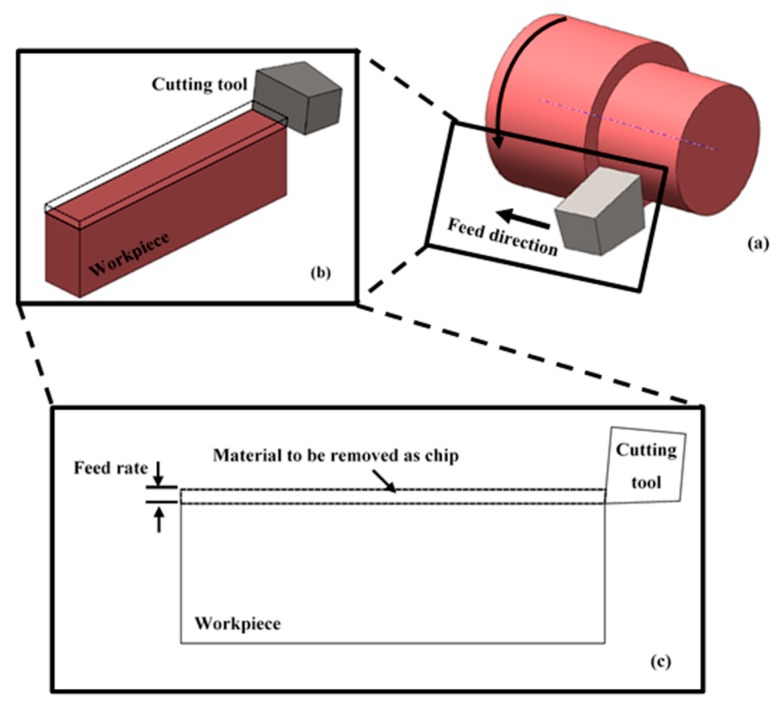
Modelling of turning process using Finite Element Method (FEM): (**a**) Turning process normal arrangement, 3D FE representation of the turning process (**b**) and (**c**) 2D FEM model of turning process where the tool remove a feed rate each stoke.

**Figure 2:**
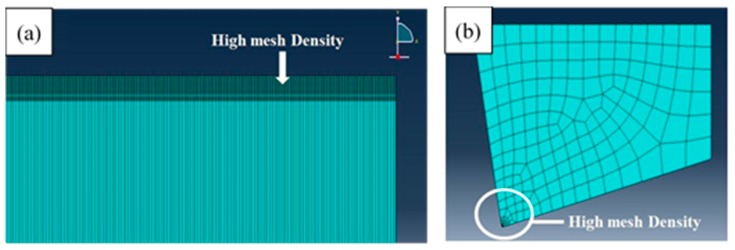
Meshing of (**a**) the workpiece and (**b**) the cutting tool.

2-D models to represent the workpiece and cutting insert were designed, see Figure 1. Plane strain quadrilateral four node reduced integration elements, CPE4R as designated in ABAQUS, were used. It was reported that although 3D FEM gave more accurate results in terms of cutting forces, 3D FEM analyses are more expensive in terms of computational time, specifically, when fine mesh is used [37]. The mesh size was chosen to give more emphasis on the chip separation region of the workpiece, which is a vital aspect during the cutting process. Therefore, the size of the elements decreased on the top area of the workpiece, and increased gradually with distance from the top surface, see Figure 2a. The same approach was employed to model the cutting tool with focus on the tip part, see Figure 2b. This helps to examine the tool wear that takes place during the cutting process; however, tool wear procreation is not part of this study.

### 2.4. Assembly

**Figure 3:**
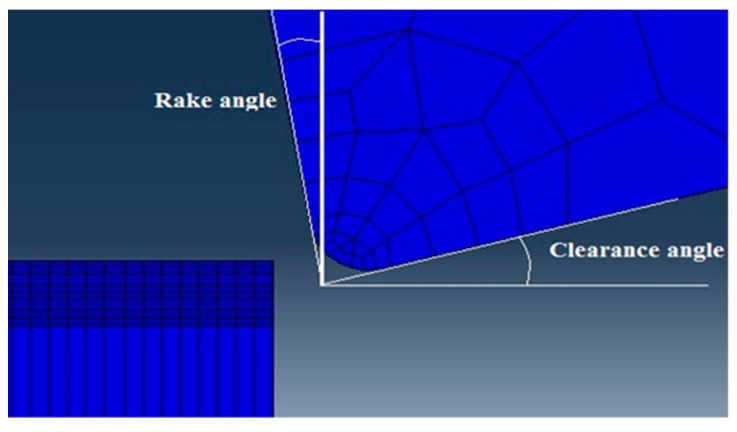
Side view of the cutting tool–workpiece assembly showing the tool angles.

In order to simulate real experimental angles and positions, the cutting tool and the workpiece were assembled together. Figure 3 depicts a side view of the assembly of the cutting tool with the workpiece.

Surface to surface contact algorithm by using penalty mechanical constraint is employed to the model. For the first surface, the tool surface is chosen and for the second surface, the workpiece surface with internal nodes, by defining a set of nodes in which the tool would engage during simulation, is chosen.

### 2.5. Boundary Conditions

**Figure 4:**
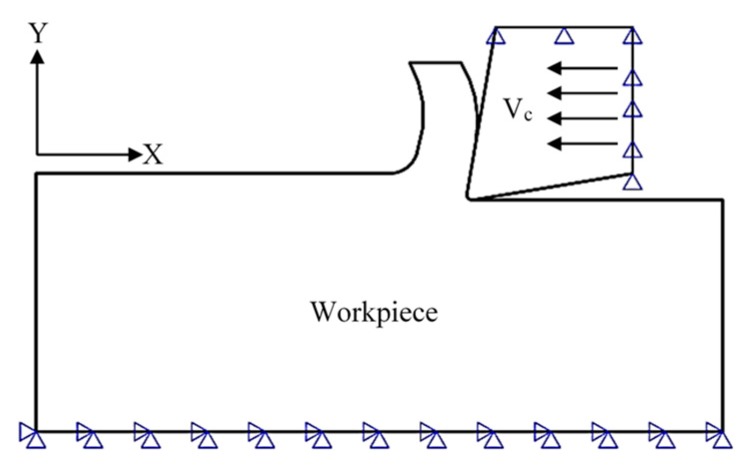
Boundary conditions for the workpiece and the cutting tool models.

The boundary conditions and data provided to Abaqus/explicit to model the cutting process are as follows. The workpiece is fixed in the X, Y, and Z-directions at its bottom surface, where the Z-direction is normal to the page. The cutting insert was fixed in the Y- and Z-directions while it was allowed to move in the X-direction with a cutting speed, V_c_, of 120 m/min which is the same as that was applied in the experimental work. Figure 4 and Table 4 summarizes the boundary conditions which were applied to both the cutting tool and the workpiece.

## 3. Experimental Validation

### 3.1. Workpiece Material

The workpiece material selected for this study was stainless steel 316L, because of its wide industrial applications and to its resistance to severe environmental conditions [38]. The workpiece material was assessed using a Spectro-lab (LAV L7) [39] to determine its chemical composition, see Table 5. Also, the hardness of the material was quantified using a Sonohard ultrasonic hardness tester, [40]. The average measured hardness was 217 ± 7 HV. It is worth stressing that although the measured hardness was not very high, stainless steel 316L is considered a hard-to-cut material, and its machining operation remains a challenging issue to be addressed [3,4]. This is because the machining of this material is associated with very tough cutting conditions owing to its superior mechanical properties, e.g., strength, toughness, wear resistance and low thermal conductivity which mean high wear of the cutting tool, thus the turning process for this material is categorized as a hard turning operation.

### 3.2. Machining Set-Up

**Figure 5:**
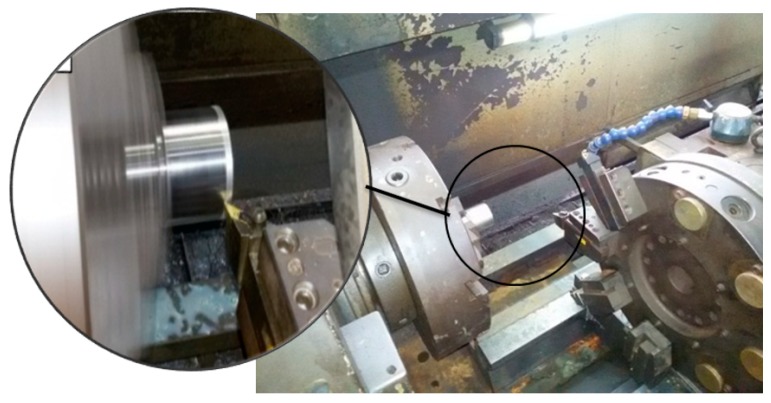
Machine setup.

Seven machining experiments were carried out. In each machining test, the machining parameters were kept the same except the feed rate which was changed from one test to the following test. The machine used for these tests was a precise three-axis computerized numerical control (CNC) turning machine, see Figure 5.

**Figure 6:**
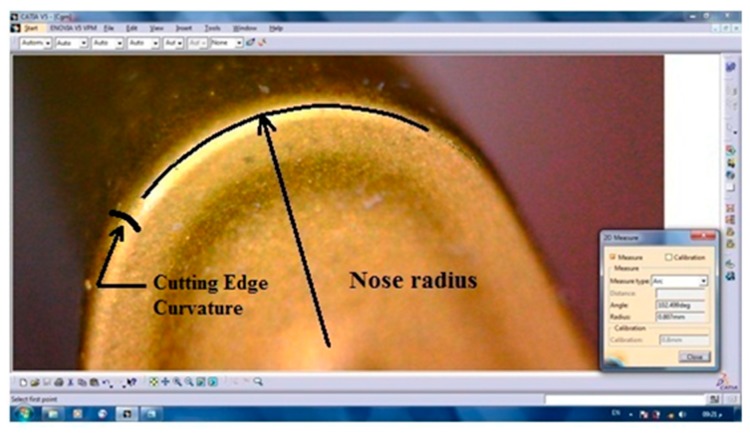
The cutting insert.

The selected cutting tool used for the experimental validation was a right-hand (CNC) turning tool. The tungsten carbide insert, SECO (VBMT 160408 TP3000), with 0.8 mm corner radius and 0.04 cutting edge radius (re), is shown in Figure 6.

### 3.3. Cutting Conditions

Due to its significant influence on the attributes of the machining process, the effect of feed rate on the performance of the precision turning operation of Stainless steel 360L (the chipping process and surface quality) was examined. In particular, the experiment was undertaken at seven different feed rates, from 10 to 70 μm/rev in steps of 10 μm. Each feed was applied to a different workpiece. As the undeformed chip thickness equals the applied feed per revolution, varying the feed per revolution made it possible to apply undeformed chip thickness values smaller and larger than the cutting edge radius of the insert. This process should be able to identify the optimum value for minimum surface roughness and proper chip formation. The remaining cutting parameters were maintained constant: cutting speed, 550 rpm (equivalent to 120 m/min), and axial depth of cut, 100 μm. The cutting conditions are listed in Table 6.

Longitudinal turning passes were carried out and repeated seven times on seven different workpieces. A digital microscope with optical magnification up to 800× was employed to image the resultant chips. A Hommel Roughness Tester T500 [41], with 4.8 mm standard tracing length and 0.8 cut-off, was utilized to quantify the surface roughness obtained. The simple arithmetic average roughness, Ra, was evaluated by averaging five readings.

## 4. Results and Discussion

### 4.1. Chip Formation: FEM Simulation vs. Experimental Results

**Figure 7:**
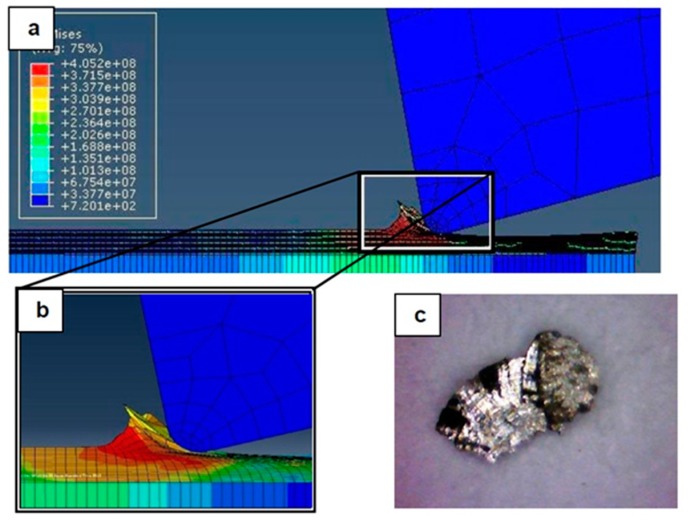
Machining Stainless Steel 316L at feed rate 0.01 mm/rev: (**a**) and (**b**) chip formation, and (**c**) produced chip.

**Figure 8:**
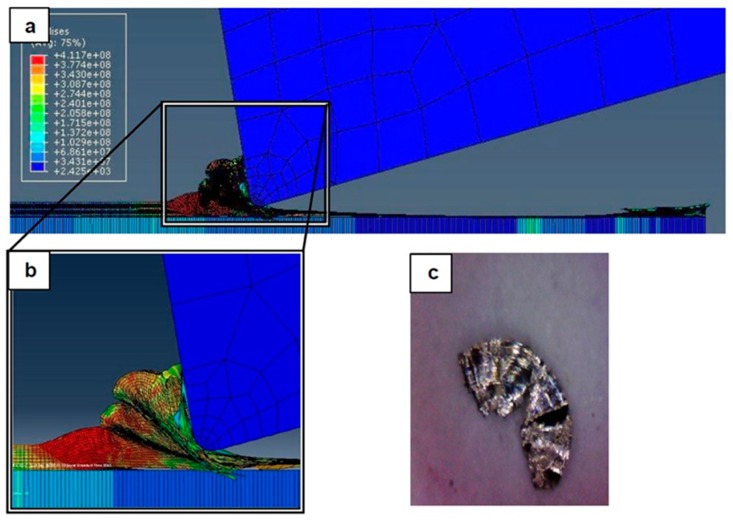
Machining Stainless Steel 316L at feed rate 0.02 mm/rev: (**a**) and (**b**) chip formation, and (**c**) produced chip.

**Figure 9:**
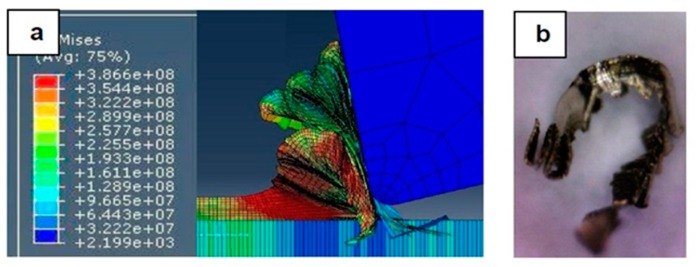
Machining Stainless Steel 316L at feed rate 0.03 mm/rev: (**a**) Chip formation and (**b**) produced chip.

**Figure 10:**
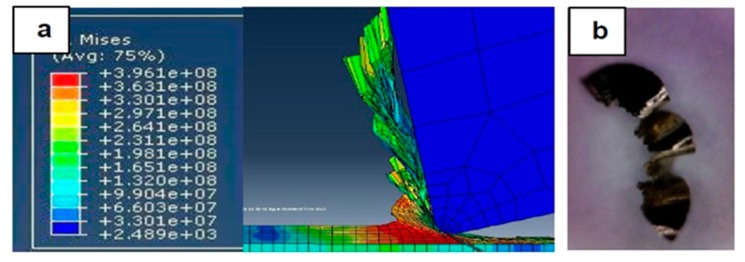
Machining Stainless Steel 316L at feed rate 0.04 mm/rev: (**a**) Chip formation and (**b**) produced chip.

**Figure 11:**
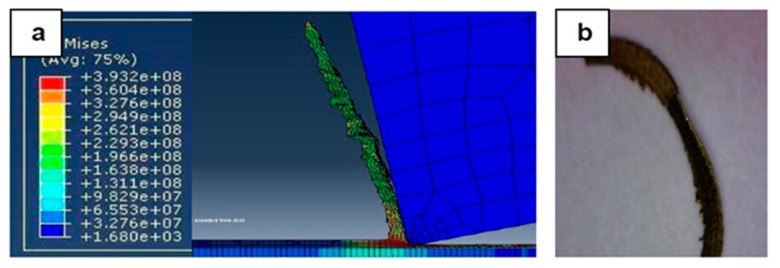
Machining Stainless Steel 316L at feed rate 0.05 mm/rev: (**a**) Chip formation and (**b**) produced chip.

**Figure 12:**
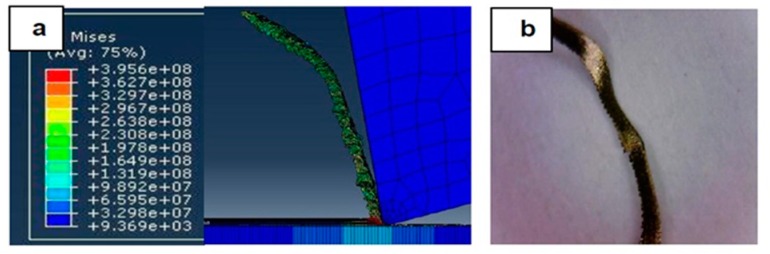
Machining Stainless Steel 316L at feed rate 0.06 mm/rev: (**a**) Chip formation and (**b**) produced chip.

**Figure 13:**
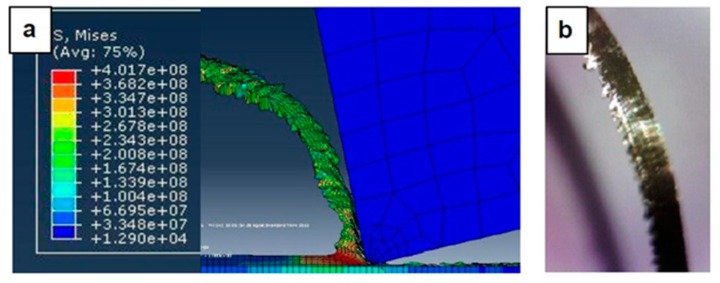
Machining Stainless Steel 316L at feed rate 0.07 mm/rev: (**a**) Chip formation and (**b**) produced chip.

Figure 7, Figure 8, Figure 9, Figure 10, Figure 11, Figure 12 and Figure 13 show chip formation obtained experimentally and using FEM simulations. It can be seen that there is an agreement between the experimental and the simulated results. Looking at the figures it is evident that the cutting process and chip formation changed significantly with the change of the applied feed rate from 0.01–0.03 mm/rev to the feed rate 0.04–0.07 mm/rev. Particularly, due to the poor cutting performance, the chip formation at the very low feed rates is not considered. It is mix of cutting and ploughing which is a result of the comparable magnitudes of the tool edge radius (Figure 6) and the feed rate. The chip formation process is governed by this relationship.

At feed rate below the minimum chip thickness; no cutting takes place while ploughing and plastic deformation of the material are the visible phenomenon. From the simulation, it was evident no chips were formed when applying a feed well below the edge radius of cutting tool, and the material is compressed underneath the cutting tool. Korkmaz et al., [42], found that no cutting takes place when the depth of cut is about 0.38 the cutting tool nose radius. At that undeformed chip thickness, or less, only plastic deformation of the uncut surface occurs and, hence, less power is consumed. When this occurs, deformed material aggregates in front of the cutting edge radius until the thickness of the material exceeded the minimum chip thickness. Then separation of the chip occurred and this process occurs repeatedly. At higher feed rates, which commences at a value in the vicinity of the tool edge radius, the chip formation process was more as expected forming continuous and longer chips. It is worth emphasizing that, at this cutting scale, the transition of both underlying regimes is very important. Particularly, it is by such critical points that the optimal cutting conditions are identified, where the minimum cutting force and highest surface quality can be obtained.

In Figure 7, the feed rate was 0.01 mm/rev. The cutting process generated a distorted chip which can be attributed to a mixed underlying mechanism, i.e., cutting and ploughing. At such low feed rate, the stresses are extremely high and rough surface due to tearing happened in the machined surface is expected. Besides, the simulation results revealed that the stress was found to be very high due to large frictional forces.

Figure 8 illustrates the simulation results at a feed rate of 0.02 mm/rev, where looking at the results obtained at this feed rate and the lower feed rate of 0.1 mm/rev, it is not so difficult to notice that the shape of the produced chip relatively changed but remained distorted. One can argue that the stresses were still very high and, again, the surface quality did not significantly improve due to the tearing that occurred in the machined surface, because the undeformed chip thickness was less than the minimum chip thickness.

In Figure 9, with the increase in the applied feed rate to 0.03 mm/rev, the shape of the generated chip was beginning to become longer, and the chip formation process was becoming more nearly stable. In addition, the stresses slightly decreased, and the effect of the minimum chip thickness became less, giving a better surface and improved cutting.

Figure 10 and Figure 11 shows the simulation results when the feed rate were 0.04 mm/rev and 0.05 mm/rev, respectively. Again, the increase in the feed rate meant that at 0.04 mm/rev, its magnitude reached the value of the tool edge radius, so the shape of the generated chip became more consistent and the stresses were noticeably reduced, and better surface quality resulted.

In Figure 12 and Figure 13, the feed rates were 0.06 mm/rev and 0.07 mm/rev. The simulation results show produced chips with a conventional formation (contentious longer chips than that obtained from lower feed rates, which was also the case with the chips produced by experiment. However, there was no substantial difference in the stress obtained and the improvement in surface quality was not so much, where it is more similar to the conventional cutting process.

### 4.2. Surface Roughness

**Figure 14:**
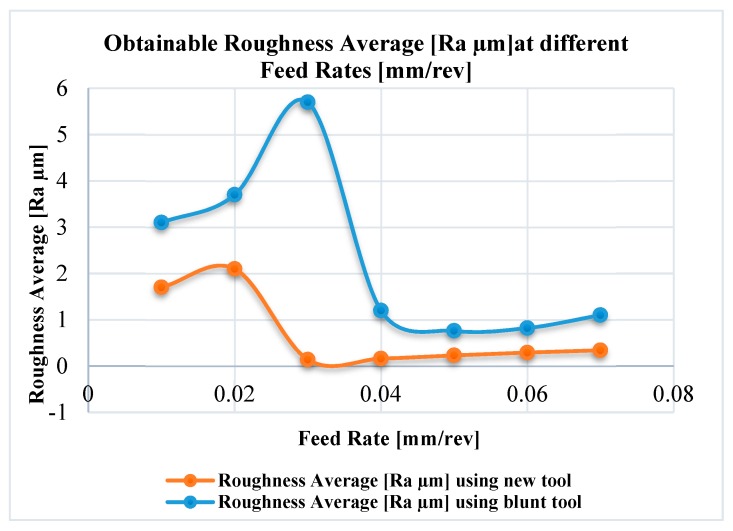
Mean surface roughness as a function of feed rate and tool wear (cutting speed 550 rpm and axial depth of cut 100 μm).

Surface roughness, Ra, was evaluated over the machined surfaces after the first and the last passes to assess the roughness generated using new and worn tools, respectively. Figure 14 shows the measured average roughness for the new and worn tools as a function of feed rate for the given conditions: Cutting speed, 120 m/min and axial depth of cut, 100 μm.

The curves shown in Figure 14 are essentially the same shape though that for the worn tool is both amplified and stretched along the abscissa compared to that for the new tool. For both new and worn tools the surface roughness, after reaching its maximum value, dropped sharply to a minimum before slowly increasing again.

From the results, it is clearly observed that, as expected, the surface roughness significantly changed with both applied feed and condition of the tool. Especially, the surface roughness generated using the worn tool was substantially greater than that produced by the new tool, but more importantly, the feed at which minimum roughness occurred increased with tool wear.

For the new tool, the minimum surface roughness (0.14 μm) was obtained at a feed rate of 0.03 mm/rev., about 0.75 re where re is the original edge radius of the new tool (0.04 mm). The maximum surface roughness (2.2 μm) occurred at a feed rate of 0.02 mm/rev., about 0.50 re. It can be also be seen that for the worn tool, the minimum surface roughness (0.81 µm) was found at a feed rate of 0.05 mm/rev, 1.25 re. Maximum surface roughness (5.7 μm) occurred at a feed rate of nearly 0.03 mm/rev, which is about 0.75 re. This can be explained by the increase of edge radius of the worn tool.

Clearly the ratio of (feed: edge radius) has an important effect on surface roughness. The curves shown in Figure 6 reflect the trade-off between kinematic parameters (cutting tool geometry and the tool trajectories) and minimum chip thickness. In particular, the decrease in surface roughness with decrease of feed rate from 0.07 mm/rev to 0.03 mm/rev (new tool) and to 0.05 mm/rev (worn tool) is due to the interaction of such kinematic factors.

The increase in roughness with decrease in feed rates below 0.03 mm/rev (new tool) and 0.05 mm/rev (worn tool) can be explained by reference to the minimum chip thickness effect, where the applied chip load below a critical value under which no cutting could take place. In this case the minimum chip thickness effect dominates the machining regime and changes the cutting mechanism into mixed cutting/ploughing which is associated with the emergence of surface defects and deterioration of the machined surface [43,44]. The reduction in mean surface roughness at very low feed rates can also be attributed to changes in the cutting mechanisms: from cutting/ploughing to burnishing [45].

It appears that for the given system, optimal levels of surface roughness will be achieved for feed rates which are numerically in the range 0.75 < (feed rate/re) < 1.25. For new tools the value of re is at the lower end, and for worn tools re is at the upper end of the range, the latter will increase in magnitude with increase of machined length, reflecting the effect of tool condition and tool wear on the achievable roughness.

## 5. Conclusions

This article has reported the results of a FEM modelling and simulation study of chip formation and surface generation during the turning of stainless steel 316L. These simulation results were then compared with those obtained by experimental trials in order to establish the feasibility of this approach. Seven sets of experimental tests were performed on a CNC turning machine and the morphologies of the formed chips were employed as the performance indicator and criteria for comparison of the FEM simulations and experimental findings. In addition, the surface roughness was experimentally evaluated twice for each machining trial, first after tuning the first path (with a new tool) and second after finishing the turning operation (where the tool considered worn) to identify the relationship between feed rate and surface roughness for new and worn tools. The main conclusions drawn are as follows:

The results reveal that a strong relationship exists between the applied feed rate, the cutting tool edge radius and the governing cutting mechanism. The chip formation process is controlled by this strong relationship. Particularly, the transition between mixed cutting/ploughing process into a complete cutting mechanism was observed when the feed rate exceeded the minimum chip thickness, meaning that the optimal cutting conditions of stainless steel 316L are at 75% of the cutting tool edge radius. Especially, when the applied feed was just greater than the value of the minimum chip thickness, the cutting mechanism altered from mixed cutting/ploughing to pure cutting with conventional chip formation. This is considered the optimal cutting parameters, when the minimum surface roughness was generated and when lowest stresses were observed.

Surface roughness was affected by feed rate in a non-linear manner. Minimum surface roughness occurs when the feed rate lies between about 0.75 to 1.25 times the original edge radius of the cutting tool. From this it is concluded that the edge radius also has an effect on surface roughness in a way that is related to feed rate. There was a major peak in the surface roughness measurements as the feed rates decreased below 0.03 mm/rev for the new tool and 0.04 mm/rev for the worn tool. This can be explained by minimum chip thickness effects. However, further reduction in the feed rate led to improvements in the surface finish which could be attributed to changes in the cutting mechanisms, from cutting to burnishing.

This investigation revealed the importance of the insert edge radius, re. The value of the cutting edge radius of the tool played an important role in the cutting process, determining the optimum feed rate to produce minimum surface roughness and optimize tool wear. Thus, re should be known before selecting a cutting insert, especially for fine turning operations where accurate dimensions and best surface finish are essential.

It is worth reiterating that this study focused on limited process parameters due to their significant influence on the process outcomes. However, in future work investigation of further process parameters such as cutting speed and type of cooling and their effects on other process responses such as cutting forces, tool wear and surface integrity will be conducted.

## Figures and Tables

**Table 1:** Parameters for the Johnson–Cook plasticity model and Johnson–Cook damage model for 316L grade stainless steel [35].

A (MPa)	B (MPa)	n	m	c	d_1_	d_2_	d_3_	d_4_
490	600	0.21	0.60	0.015	0.05	3.44	2.12	0.002

**Table 2:** Mechanical properties of 316L grade stainless steel [36].

Grade	Tensile Strength (MPa) Min	Yield Strength 0.2% Proof (MPa) Min	Elongation (% in 50 mm) Min	Hardness Hv	
				Rockwell Max	Vickers (HV)
316L	515	205	40	95	222

**Table 3:** Physical properties of 316L grade stainless steel [36].

Grade	Density kg/m^3^	Elastic Modulus GPa	Specific heat 0–100 °C J/kg °C
316L	8000	193	500

**Table 4:** Boundary conditions for the modelling of the workpiece and the cutting tool.

Boundary Condition	Workpiece	Cutting Tool
Displacement in *x* axis = U_X_	0	−150 mm
Displacement in *y* axis = U_Y_	0	0
Displacement in *z* axis = U_Z_	0	0
Velocity	0	120 m/min in negative X axis direction

**Table 5:** Chemical composition of stainless-steel 316l (in weight percent wt%).

C	Mn	Si	Cr	Co	Ni	Mo	Fe
0.03	0.95	0.5	15.7	0.19	10	2.2	Balance

**Table 6:** Cutting conditions.

Constant Parameters	Changed Parameters
Workpiece diameter (D) = 70 mm	Feed rate for experiment 1 = 0.01 mm/rev
Workpiece length = 80 mm	Feed rate for experiment 2 = 0.02 mm/rev
Lathe chuck revolutions/min (n)= 550 rpm	Feed rate for experiment 3 = 0.03 mm/rev
Cutting length (L) = 50 mm	Feed rate for experiment 4 = 0.04 mm/rev
Depth of cut (ap) = 0.01 mm	Feed rate for experiment 5 = 0.05 mm/rev
	Feed rate for experiment 6 = 0.06 mm/rev
	Feed rate for experiment 7 = 0.07 mm/rev
